# Burden of Dengue in Pregnant Individuals: A Systematic Literature Review

**DOI:** 10.4269/ajtmh.25-0311

**Published:** 2026-04-21

**Authors:** Rada Rusu, Anna Durbin, Isabella Clements, Hannah Frost, Danielle Mariner-Goff, Katherine Massey, Molly Murton, Shirley V. Sylvester

**Affiliations:** ^1^ Johnson & Johnson, Toronto, Canada;; ^2^ John Hopkins Bloomberg School of Public Health, Baltimore, Maryland, USA;; ^3^ Costello Medical Consulting Ltd, Manchester, United Kingdom;; ^4^ Costello Medical Consulting Ltd, Cambridge, United Kingdom;; ^5^ Costello Medical Singapore Pte Ltd, Singapore;; ^6^ Johnson & Johnson, Bern, Switzerland

## Abstract

Many infectious diseases are known to have increased severity among pregnant people, yet limited data exist on the burden of dengue in this population. This systematic review collated studies conducted from 2010 onward to capture epidemiological data and a variety of pregnancy outcomes of those infected with dengue during gestation. Among the 23 included publications, dengue infections in pregnant individuals were most frequently contracted in the third trimester. Dengue-infected mothers appear to be associated with a higher risk of maternal mortality (0–21.7%), pregnancy loss (0–11.6%), and preterm birth (4.3–37.5%) compared with healthy mothers. However, heterogeneity of data, missing quantitative comparisons of outcomes in dengue-positive and dengue-negative pregnant people, and lack of standardization of study methods and measurement emphasize an evidence gap. As dengue infections continue to rise and transmission boundaries expand, it is imperative to include pregnant people in dengue studies, surveillance, and public health measures.

## INTRODUCTION

Dengue is a viral infection transmitted by *Aedes aegypti* mosquitos and caused by dengue virus (DENV), of which there are four serotypes (DENV1, -2, -3, and -4).^1^^,^^2^ Dengue is endemic to more than 100 tropical and subtropical countries, with about 100–400 million dengue infections occurring annually.^2^ Estimated incidence of dengue cases has doubled every 10 years since 1990, putting about half of the world’s population at risk of dengue.^3^ Most dengue cases are asymptomatic; however, one in four patients shows symptoms of fever, severe headache nausea, vomiting, and muscle and joint pain.^4^ In addition, patients can develop severe dengue,^5^ which in rare cases, can lead to death.^2^

A pregnant individual’s immune system is in constant flux to ensure that the fetus is not rejected.^6^ Changes in immune system function can lead to increased susceptibility to some infectious diseases.^7^ Evidence on the burden of dengue infection during pregnancy is limited; however, contracting dengue during pregnancy has been shown to have harmful effects on pregnant individuals.^8^ Pregnant individuals have a higher risk of developing severe dengue as compared with nonpregnant individuals.^9^ Additionally, dengue infection during pregnancy may result in preterm birth, low birth weight, fetal distress, and increased risk of fetal death.^10^^,^^11^ Furthermore, evidence suggests that vertical transmission between mother and baby is possible, perhaps linked to the timing of infection during pregnancy.^11^ Nonetheless, policies that exclude pregnant individuals from clinical trial research because of their vulnerability have led to this population remaining underrepresented in dengue research.^12^

Given that DENV’s transmission will likely increase by 37% in 2050,^13^ the incidence of dengue among pregnant people will also rise. As such, it is important to proactively understand the disease progression among the populations most at risk, such as pregnant individuals, to effectively and appropriately care for those who may become infected or to include them in intervention clinical trials. This systematic literature review (SLR) was conducted with the objective of characterizing the burden of dengue infection among pregnant individuals and in fetuses, newborns, and infants up to 1 year of age born to dengue-infected mothers. The focus of this article is on outcomes in pregnant individuals; evidence concerning neonatal outcomes and vertical transmission can be found in Supplemental Text.

## MATERIALS AND METHODS

This review was conducted in accordance with a prespecified protocol (CRD42024493937) and with stringent methodological principles of conduct for SLRs.^14^^–^^16^

### Search strategy.

Electronic databases (MEDLINE databases, Embase, and the Cochrane Database of Systematic Reviews) were searched from 2010 to November 21, 2023 using search terms for dengue and pregnancy. Proceedings from the last 2 years (2021–2023) of key congresses (the Asia Dengue Summit, the International Congress on Infectious Diseases, ID Week, PanDengue, and the American Society of Tropical Medicine and Hygiene Annual Meeting), non-English databases, the WHO International Clinical Trials Registry Platform, WHO and CDC websites, and the bibliographies of identified SLRs and (network) meta-analyses were additionally hand searched. Full details of the literature searches, including search strategies, are available in Supplemental Text and Supplemental Tables 1–3.

### Study selection.

Studies were screened against prespecified eligibility criteria based on the Population, Intervention, Comparator and Outcomes framework (Table 1) by two independent reviewers at both the title/abstract and full-text review stages, with discrepancies resolved through discussion or adjudication by a third reviewer. Supplemental Figures 1 and 2 show the selection strategy during both title/abstract and full-text review stages.

**Table 1:** Eligibility criteria for the systematic literature review

Domain	Inclusion Criteria	Exclusion Criteria
Patient population	Pregnant individuals infected with dengue virus diagnosed through a laboratory test Newborns up to 1 year of age born from someone infected with dengue virus	Children or adults without diagnosed dengue Adults with dengue who are not pregnant Newborns who are not born from someone infected with dengue virus
Intervention/comparator	None or supportive care	Any experimental intervention to treat dengue
Outcomes	Prevalence/incidence Hospitalization ICU/NICU admission rates Mortality rates Disease severity among those with an initial or repeat infection (nonsevere vs. severe) Pregnancy outcomes Preterm births Spontaneous abortions Stillbirths Miscarriage/fetal loss IUGR Pre-eclampsia Postpartum hemorrhage Interpregnant hemorrhage Fetal/newborn outcomes (up to 1 year of age) Birth weight 28-day neonatal mortality Infant mortality Major congenital abnormalities SGA Vertical transmission incidence	Any other outcomes (e.g., costs, effectiveness of preventive measures, test accuracy, and patient perspectives)
Study design	Observational and real-world studies (cohort, case–control, cross-sectional, and chart review) Routinely collected surveillance data	Any other study type, including interventional studies and case reports
	Relevant SLRs, NMAs, and MAs were eligible at the title/abstract review stage and hand searched for relevant primary studies, but they were excluded during the full-text review stage unless they reported primary research	
Language	Any language*	NA
Other	Studies published from 2010 onward Any country Conference abstract published in or after 2021	Studies published before 2010 Congress abstracts published before 2021

ICU = intensive care unit; IUGR = intrauterine growth restriction; MA = meta-analyses; NA = not applicable; NICU = neonatal intensive care unit; NMA = network meta-analyses; SGA = small for gestational age; SLR = systematic literature review.

* Google Translate software was used to aid the review of non-English sources.

### Data extraction and quality assessment.

From a subset of the highest-priority studies (those with sample size of ≥30 and reporting on at least two outcomes of interest), key information (study characteristics, patient baseline characteristics, epidemiological outcomes, and outcomes in pregnant individuals) was extracted by a single reviewer into a predefined data extraction grid and independently verified by a second reviewer. Discrepancies were resolved through discussion or adjudication by a third individual. In some cases, simple calculations were performed based on data available in the study.

The quality of all included studies was assessed by one reviewer and verified by a second individual using the Alberta Heritage Foundation for Medical Research (AHFMR) tool.^17^

**Figure 1.**
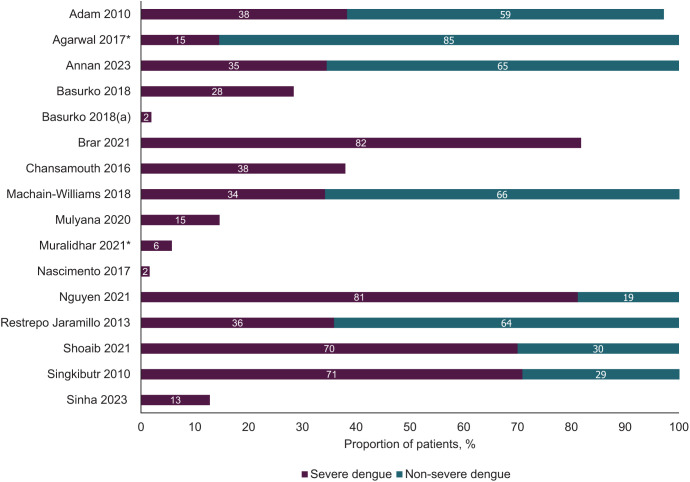
Broad categorization of dengue severity across extracted studies. Data were rounded to zero decimal places, consistent with the highest level of granularity across all included studies. Studies report dengue severity for all dengue-positive pregnant people unless otherwise indicated. *Percentages were manually calculated from the data available within the study.

Because of observed inconsistencies in the definitions of “severity” among studies and to enable comparisons, definitions that described acute symptoms of dengue (dengue hemorrhagic fever [DHF], dengue shock syndrome, “dengue with warning signs” [WS], and “severe”) were grouped into a broad “severe” category. Similarly, definitions describing mild or no symptoms (“dengue” and “dengue without WS”) were grouped into a “nonsevere” category (Figure 1). This categorization of severe dengue and nonsevere dengue is used throughout this article.

## RESULTS

### Searches and screening.

**Figure 2.**
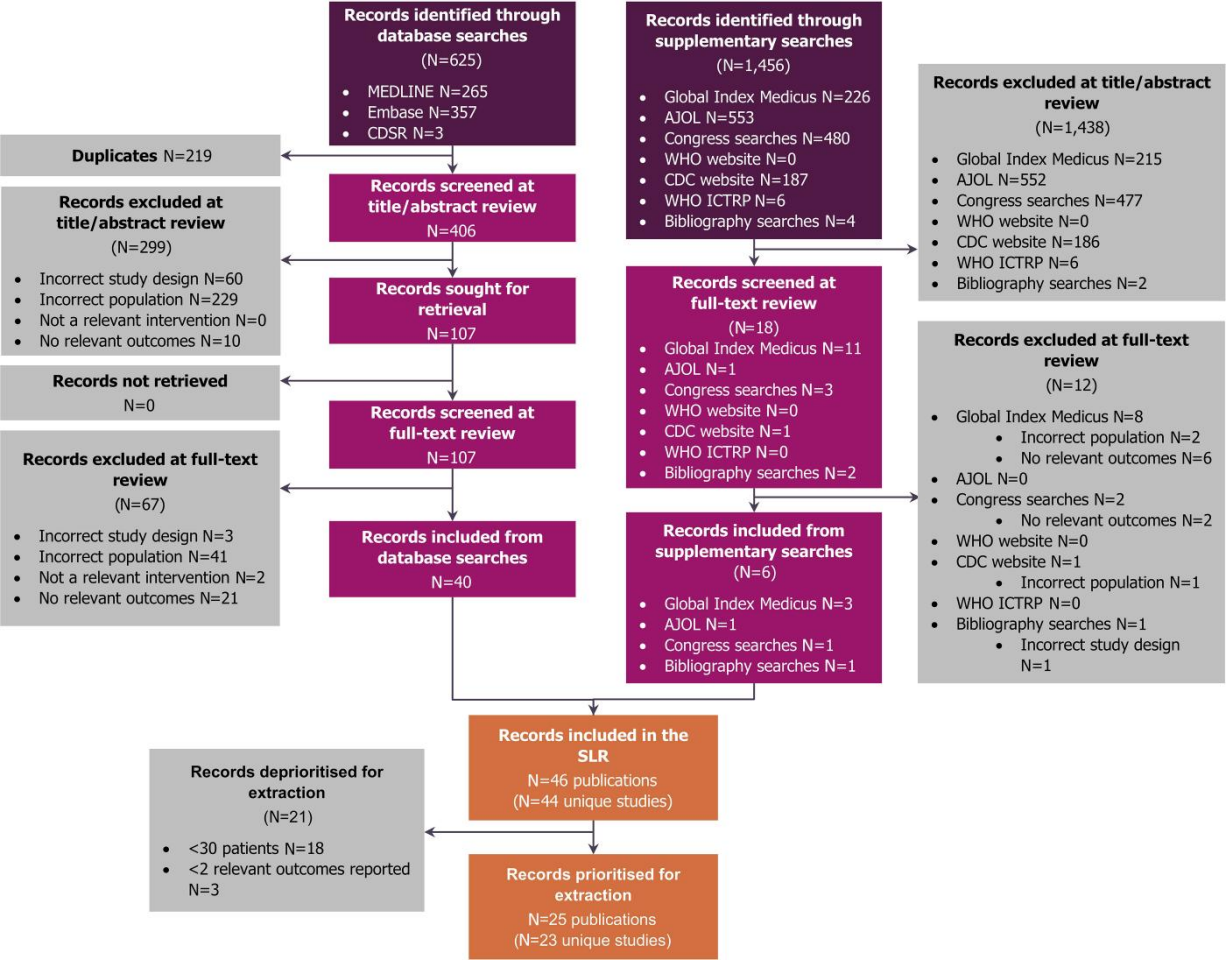
Preferred Reporting Items for Systematic Reviews and Meta-Analyses flow diagram. AJOL = African Journals Online; CDSR = Cochrane Database of Systematic Reviews; ICTRP = International Clinical Trials Registry Platform; SLR = systematic literature review.

A Preferred Reporting Items for Systematic Reviews and Meta-Analyses diagram displaying the flow of records through each stage of the SLR is presented in Figure 2. Through the electronic database searches, a total of 625 publications were identified. After the removal of duplicates, 406 title/abstracts were reviewed followed by 107 full-text publications, and 40 publications were ultimately included. Six further records were included from the supplementary searches, resulting in a total of 46 publications. Of these, 23 unique studies included a sample size of ≥30 patients and reported at least two outcomes in dengue-positive pregnant people. These studies were explored within this review.

### Study characteristics.

**Figure 3.**
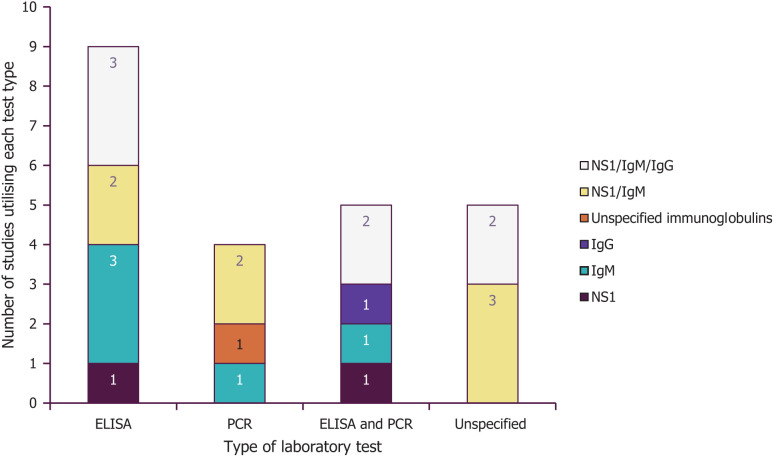
Types of laboratory testing in the extracted studies. NS1 = nonstructural protein 1; PCR = polymerase chain reaction.

Most studies were retrospective cohort analyses and conducted at a single center (*n* = 12). The studies were conducted in 14 different countries across three continents: Asia (*n* = 14), Latin America and the Caribbean (*n* = 7), and sub-Saharan Africa (*n* = 3). All study characteristics are presented in Table 2.^18^^–^^40^ The most common laboratory test was ELISA (Figure 3).^18^^–^^31^ ELISA testing most frequently focused on the detection of Immunoglobulin M (IgM) or a combination of nonstructural protein 1, IgM, and Immunoglobulin G (IgG).

**Table 2:** Study characteristics of extracted studies

Study Name	Country	Sample Size	Study Design	Population Source	Primary Population	Laboratory Tests Used to Confirm Dengue	Relative Quality Ranking*
Africa							
Tougma et al.^38^	Burkina Faso	121	Retrospective cohort	Regional sample; Yalgado Ouedraogo University Teaching Hospital, Tingandogo University Teaching Hospital, Bogodogo District Hospital, Saint Camille Hospital, Medical Center with Surgical Services Schiphra and the Medical Center with Surgical Services Paul VI	Dengue-positive pregnant people	Rapid diagnosis test SD Bioline Dengue Duo (NS1/IgM/IgG)	2
Smith et al.^26^	Kenya	35	Prospective cohort	NR	Dengue-positive pregnant people	ELISA and PCR (IgG)	4
Adam et al.^18^	Sudan	78	Retrospective cohort	Regional sample; PortSudan and Elmawani hospitals	Dengue-positive pregnant people	ELISA (IgM)	2
Asia							
Garg et al.^21^	India	196	Prospective cohort	Regional sample; Sarojini Naidu Medical College and three private hospitals in India	Dengue-positive pregnant people	ELISA (NS1/IgM)	4
Kaur and Singh^22^	India	100	Prospective cohort	NR	Dengue-positive pregnant people	ELISA (IgM)	4
Sagili 2022^39^	India	91	Retrospective cohort	Single hospital; Jawaharlal Institute of Postgraduate Medical Education and Research	Dengue-positive pregnant people	Unspecified (NS1/IgM)	2
Agarwal et al.^34^	India	62	Retrospective cohort	Single hospital; Safdarjung Hospital	Dengue-positive pregnant people	Unspecified (NS1/IgM)	2
Sinha and Datta^37^	India	62	Retrospective cohort	Single Hospital; Jamshedpur in Jharkand, India	Dengue-positive pregnant people	PCR (NS1/IgM)	3
Brar et al.^20^	India	44	Prospective cohort	Single hospital; Post Graduate Institute of Medical Education and Research	Dengue-positive pregnant people	ELISA (NS1)	4
Muralidhar et al.^24^	India	35	Retrospective cohort	Single hospital; Vydehi Institute of Medical Sciences and Research Center	Dengue-positive pregnant people	ELISA (NS1/IgM/IgG)	4
Mulyana et al.^23^	Indonesia	41	Prospective cohort	Single hospital; Sanglah General Hospital	Dengue-positive pregnant people	ELISA (NS1/IgM/IgG)	4
Chansamouth et al.^28^	Laos	76	Prospective cohort	Single hospital; Mahosot Hospital	Dengue-positive pregnant people	ELISA and PCR (NS1/IgM/IgG)	3
Shoaib et al.^36^	Pakistan	50	Prospective cohort	Single hospital; Department of Obstetrics & Gynecology, Sandeman Provincial Hospital	Dengue-positive pregnant people	Unspecified (NS1/IgM/IgG)	2
Mubashir et al.^35^	Pakistan	48	Retrospective cohort	Single hospital; Aga Khan University Hospital	Dengue-positive pregnant people	PCR (NS1/IgM)	3
Singkibutr et al.^33^	Thailand	48	Retrospective cohort	Single hospital; Sunpasitthiprasong Hospital	Dengue-positive pregnant people	Unspecified (NS1/IgM/IgG)	3
Nguyen et al.^25^	Vietnam	32	Retrospective cohort	Single hospital; Children’s Hospital 1, Ho Chi Minh, Vietnam	Infants and newborns born of dengue-positive mothers	ELISA (NS1/IgM)	2
South America							
López Barroso et al.^19^	Cuba	30	Retrospective cohort	NR	Dengue-positive pregnant people	ELISA (IgM)	4
Annan et al.^32^	Mexico	4,943	Cross-sectional	National sample; Mexico’s Ministry of Health	Dengue-positive pregnant people	PCR (unspecified)	1
Machain-Williams et al.^40^	Mexico	82	Retrospective cohort	Regional sample; n9 public hospitals located in 3 states (3 hospitals per state)	Dengue-positive pregnant people	Unspecified (NS1/IgM)	2
Nascimento et al.^30^	Brazil	3,898	Retrospective cohort	National sample; Brazilian national reportable disease information system database; live birth information system database	Dengue-positive pregnant people	ELISA and PCR (IgM)	1
Restrepo Jaramillo et al.^31^	Colombia	39	Prospective cohort	NR	Dengue-positive pregnant people	PCR (IgM)	3
Friedman et al.^29^	French Guiana	86	Retrospective cohort	Single hospital; Franck Joly Hospital	Infants and newborns born of dengue-positive mothers	ELISA and PCR (NS1)	1
Basurko et al.^27^	French Guiana	73	Prospective cohort	Regional; participating (hospitals, mother-and-child care centers, and private practitioners in French Guiana)	Dengue-positive pregnant people	PCR (NS1/IgM/IgG)	1

NR = not reported; NS1 = nonstructural protein 1; PCR = polymerase chain reaction.

* Study quality was assessed using the Alberta Heritage Foundation for Medical Research checklist. A relative ranking was assigned based on the number of questions that were fully, partially, or not addressed. A score of one indicates that all questions were fully addressed. A score of two indicates that one question was not addressed and that one or two questions were partially addressed. A score of three indicates that two questions were not addressed and that zero to one question was partially addressed or that one question was not addressed and three questions were partially addressed. A score of four indicates that two questions were not addressed and that two to three questions were partially addressed or that three questions were not addressed.

### Characteristics of dengue infections.

**Figure 4.**
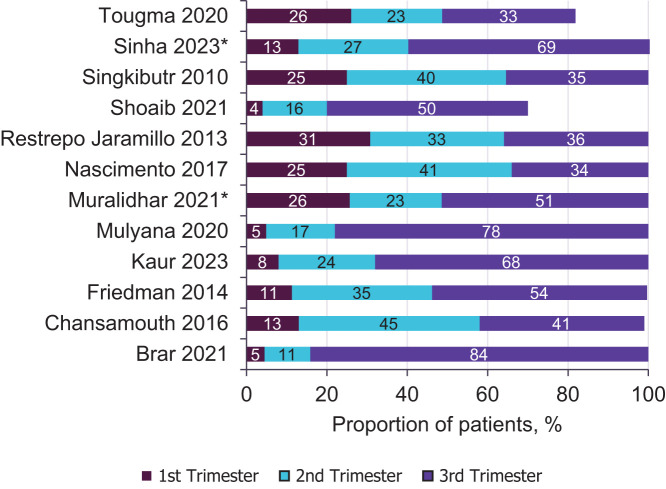
Trimester of infection across all extracted studies. Data were rounded to zero decimal places, consistent with the highest level of granularity across all included studies. *Percentages were manually calculated from the data available within the study.

Four studies reported on dengue infection serotype (Supplemental Figure 3).^27^^,^^28^^,^^31^^,^^32^ Dengue virus-2 was the most frequently reported serotype (*n* = 4), whereby the reporting studies were conducted in Mexico,^32^ French Guiana,^27^ Laos,^28^ and Colombia.^31^ The proportion of patients with DENV-2 ranged from 2.6%^31^ to 97.0%.^27^ Fifteen studies specified severity of dengue infection (Figure 1), whereas four studies^22^^,^^23^^,^^27^^,^^33^ reported on dengue immunity among infected mothers; secondary infections ranged from 20.4%^22^ to 90.2%.^23^ Another 15 studies reported on the time point of dengue infection; the mean or median time point of infection ranged from 20 to 34 weeks of gestation.^18^^,^^20^^,^^22^^–^^24^^,^^28^^–^^31^^,^^33^^–^^38^ Of the 12 studies reporting by trimester of pregnancy, the third trimester was the most frequently reported time point of infection, with the proportion of patients ranging from 33.2%^38^ to 84.1%^20^ (Figure 4). Details regarding the age and ethnicity of study participants can be found in Supplemental Text and Supplemental Figure 4.

### Study outcomes.

#### Epidemiological outcomes.

Epidemiological outcomes were not frequently reported (Table 3). No studies reported incidence or general hospitalization rate. A single high-quality study by Adam et al.^18^ reported period prevalence of dengue in eastern Sudan between 2008 and 2009 and found dengue in 0.7% (0.4% nonsevere cases and 0.3% severe cases) of 10,820 deliveries across two hospitals. The work of Annan et al.,^32^ another high-quality study, reported the proportion of dengue cases across Mexico from 2012 to 2020. A total sample of 94,832 dengue cases were found in women, of which 4,943 (5.2%) were pregnant (65.5% nonsevere dengue and 34.6% severe dengue). Annan et al.^32^ reported a significantly higher proportion of severe dengue within the pregnant population than *the* nonpregnant population (34.5% versus 25.9%, respectively; *P* <0.0001).^32^ Six studies specifically recruited a population of patients who were hospitalized,^24^^,^^28^^,^^33^^,^^35^^,^^38^^,^^39^ but none investigated the rate of hospitalization among pregnant women with dengue. Four studies reported the proportion of intensive care unit (ICU) admission among pregnant women with dengue,^24^^,^^35^^,^^36^^,^^40^ and admission rates ranged widely from 5.7%^24^ to 60.0%.^40^ As the studies’ data were heterogeneous, the association between dengue and ICU admission among the pregnant population was inconclusive. Of note, clinical signs of dengue infection in mothers were reported in 16 manuscripts,^18^^–^^20^^,^^22^^–^^25^^,^^27^^,^^31^^,^^33^^–^^37^^,^^39^^,^^40^ with thrombocytopenia,^20^^,^^22^^–^^24^^,^^33^^,^^36^^,^^39^ fever,^25^^,^^36^^,^^37^^,^^40^ pleural effusions,^31^^,^^39^^,^^40^ ascites,^31^^,^^39^^,^^40^ epistaxis,^18^^,^^31^^,^^34^ and headache,^31^^,^^37^^,^^40^ as the most reported signs among dengue-infected mothers. Seven studies reported laboratory abnormalities,^22^^,^^27^^,^^31^^,^^33^^,^^34^^,^^37^^,^^40^ of which elevated alanine aminotransferase and aspartate transaminase levels were most often reported.^27^^,^^34^^,^^37^^,^^40^

**Table 3:** Summary of the reporting of study outcomes in extracted studies

Outcomes	No. of Studies Reporting (*n*)*
Epidemiology outcomes	
Incidence	0
Prevalence	1
Hospitalization rate	0
ICU admissions	4
NICU admissions	8
Pregnancy outcomes	
Maternal mortality	14
Pregnancy loss	13
Preterm birth	20
Intrauterine growth restriction	5
Pre-eclampsia	7
Delivery	18
Postpartum hemorrhage	10
Infant outcomes	
Neonatal mortality	7
Birth weight	14
Small for gestational age	2
Congenital abnormalities	3
Vertical transmission	7

ICU = intensive care unit; NICU = neonatal intensive care unit.

* The frequency outcomes reported across included studies are indicated: not reported (0 studies), moderately reported (1–9 studies), and frequently reported (≥10 studies).

#### Maternal mortality.

Maternal mortality was reported by 14 studies.^18^^,^^20^^,^^24^^,^^25^^,^^28^^,^^31^^,^^33^^–^^40^ The rate of maternal mortality among all dengue-positive pregnant individuals ranged from 0.0%^24^^,^^25^ to 21.7%.^18^ Adam et al.^18^ reported the highest rate of maternal mortality; of 78 pregnant women with confirmed dengue IgM serology, 17 (21.7%) died, all with severe dengue.^18^ The studies of Muralidhar et al.^24^ and Nguyen et al.,^25^ which reported the lowest proportions of maternal mortality (zero deaths), were judged to be of the lowest quality and moderate quality, respectively (Table 4).^24^^,^^25^ The work of Muralidhar et al.^24^ was based in India and included 35 patients, of which a low proportion (5.7%) met the criteria for DHF (defined by WHO 2009 guidance). The work of Nguyen et al.^25^ was conducted within a hospital setting in Vietnam, and it included 32 patients; maternal recruitment was a secondary recruitment process.

**Table 4:** Summary of study quality as assessed using the Alberta Heritage Foundation for Medical Research tool

Study	1. Question/ Objective	2. Study Design	3. Participant Selection	4. Patient Characteristics	8. Outcomes and Exposures	9. Sample Size	10. Analytic Methods	11. Estimate of Variance	12. Confounding	13. Reporting	14. Conclusions	Relative Ranking*
Annan et al.^32^	Y	Y	Y	Y	Y	Y	Y	Y	Y	Y	Y	1
Basurko et al.^27^	Y	Y	Y	Y	Y	Y	Y	Y	Y	Y	Y	1
Friedman et al.^29^	Y	Y	Y	Y	Y	Y	Y	Y	Y	Y	Y	1
Nascimento et al.^30^	Y	Y	Y	Y	Y	Y	Y	Y	Y	Y	Y	1
Adam et al.^18^	Y	Y	Y	Y	Y	Y	Y	Y	N	Y	Y	2
Machain-Williams et al.^40^	Y	Y	Y	Y	Y	Y	Y	Y	N	Y	Y	2
Nguyen et al.^25^	Y	Y	Y	Y	Y	P	Y	Y	N	Y	Y	2
Tougma et al.^38^	Y	Y	Y	Y	P	Y	Y	N	Y	Y	Y	2
Agarwal et al.^34^	Y	Y	Y	Y	P	P	Y	Y	N	Y	Y	2
Sagili et al.^39^	Y	Y	Y	Y	P	P	Y	Y	N	Y	Y	2
Shoaib et al.^36^	Y	Y	Y	Y	Y	P	P	Y	N	Y	Y	2
Chansamouth et al.^28^	Y	Y	Y	Y	Y	Y	Y	Y	N	Y	N	3
Mubashir et al.^35^	Y	Y	Y	Y	N	P	Y	Y	N	Y	Y	3
Restrepo Jarmillo et al.^31^	Y	Y	Y	Y	P	Y	Y	N	N	Y	Y	3
Singkibutr et al.^33^	Y	Y	Y	Y	P	P	Y	N	P	Y	Y	3
Sinha and Datta^37^	Y	Y	Y	Y	P	P	Y	Y	N	Y	P	3
López Barroso et al.^19^	Y	Y	Y	P	P	Y	Y	N	N	Y	Y	4
Brar et al.^20^	Y	Y	Y	Y	P	P	N	Y	N	Y	Y	4
Garg et al.^21^	Y	Y	Y	P	P	Y	N	P	N	Y	Y	4
Mulyana et al.^23^	Y	Y	Y	P	P	P	N	N	N	Y	Y	4
Muralidhar et al.^24^	Y	Y	Y	P	P	P	N	N	N	Y	Y	4
Kaur and Singh^22^	Y	P	Y	P	P	P	N	N	N	Y	Y	4
Smith et al.^26^	Y	P	Y	P	P	P	N	N	N	Y	Y	4

Studies are ordered from highest to lowest quality as assessed using the Alberta Heritage Foundation for Medical Research tool for quantitative studies. Studies judged to fully address a question were awarded a “Y.” Studies that partially addressed a question but lacked some detail or clarity were awarded a “P.” Studies that did not address a question or severely lacked detail or clarity were awarded an “N.”

* A relative ranking was assigned based on the number of questions that were fully, partially or not addressed. A score of one indicates that all questions were fully addressed. A score of two indicates that one question was not addressed and that one or two questions were partially addressed. A score of three indicates that two questions were not addressed and that zero to one question was partially addressed or that one question was not addressed and three questions were partially addressed. A score of four indicates that two questions were not addressed and that two to three questions were partially addressed or that three questions were not addressed.

One study by Tougma et al.^38^ compared the rate of maternal mortality in dengue-positive and dengue-negative groups of hospitalized febrile pregnant people in a regional sample of Burkina Faso and noted that maternal death was statistically associated with dengue. Machain-Williams et al.^40^ reported the numbers of maternal deaths of dengue-positive pregnant people from a regional sample in Mexico in three subgroups: dengue with WS, dengue without WS, and severe dengue. The subgroups of dengue with and without WS experienced no maternal mortality. However, 5 of 13 patients (38.5%) with severe dengue died, all because of organ failure during or after emergency cesarean section (C-section) operations as a result of acute fetal distress.^40^ In a single-hospital analysis in India, Sinha and Datta^37^ compared cases of maternal deaths among pregnant people infected with dengue during late pregnancy versus early pregnancy (gestation >24 weeks versus <24 weeks, respectively). Four of 32 patients (12.5%) infected in late pregnancy died, whereas death was not reported among those infected during early pregnancy.^37^

#### Pregnancy outcomes.

Pregnancy outcomes were most frequently reported, with most outcomes noted in ≥10 of the included studies (Table 3). Preterm birth was the most commonly reported pregnancy outcome across included studies (*n* = 20)^18^^–^^31^^,^^33^^,^^34^^,^^36^^,^^38^^–^^40^ followed by birth type (*n* = 18)^18^^,^^20^^,^^21^^,^^23^^–^^27^^,^^29^^–^^31^^,^^33^^–^^36^^,^^38^^–^^40^ and maternal mortality (*n* = 14).^18^^,^^20^^,^^24^^,^^25^^,^^28^^,^^31^^,^^33^^–^^40^

**Figure 5.**
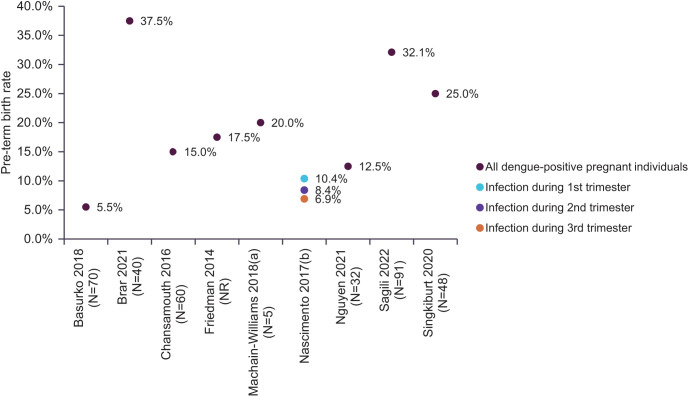
Rate of preterm birth defined as birth at <37 weeks of gestational age across extracted studies. NR = not reported.

Among the 20 studies reporting on preterm birth, the most commonly used definition was <37 weeks of gestational age (9 studies) (Figure 5).^20^^,^^25^^,^^27^^–^^30^^,^^33^^,^^39^^,^^40^ The rate of preterm birth in dengue-infected pregnancies ranged from 4.3%^27^ to 37.5%.^20^ Seven studies investigated the statistical association of dengue with preterm birth rates,^19^^,^^22^^,^^26^^,^^27^^,^^33^^,^^38^^,^^39^ of which only three studies (those of López Barroso et al.,^19^ Singkibutr et al.,^33^ and Tougma et al.^38^) reported a statistically significant difference between dengue-positive and dengue-negative pregnant people. Nascimento et al.^30^ explored the relationship between the rate of preterm birth (gestation <37 weeks) and the time point of dengue infection. The study noted that preterm birth decreased as the trimester of infection increased. However, when comparing with pregnant women randomly selected from live-born children, dengue infection in the third trimester increased the likelihood of women experiencing preterm birth (adjusted odds ratio [OR]: 1.24, 95% CI: 0.97–1.6, *P* = 0.08).^30^ In a high-quality study, Agarwal et al.^34^ also reported a high rate of preterm birth among those infected in the third trimester of pregnancy. This study found that 41.0% of dengue-positive pregnant people diagnosed at 26–36 weeks of infection experienced preterm birth (*n* = 9/22).

Rates of vaginal delivery, C-section, and emergency C-section delivery were reported in 11 studies,^20^^,^^21^^,^^23^^–^^25^^,^^29^^–^^31^^,^^33^^,^^35^^,^^40^ 17 studies,^18^^,^^20^^,^^21^^,^^23^^–^^27^^,^^29^^–^^31^^,^^33^^–^^36^^,^^38^^,^^39^ and 2 studies,^27^^,^^40^ respectively. No trend was observed for any type of birth (vaginal, C-section, or emergency C-section). Each delivery type had highly heterogeneous results, particularly vaginal births, which ranged from 19.5%^23^ to 90.6%.^25^ No significant differences in C-section or emergency C-section rates between dengue-positive and dengue-negative people were reported.

Pregnancy loss, which was defined as any of spontaneous abortion, stillbirth and miscarriage, or death in utero (DIU), within dengue-positive pregnant people was reported in 13 studies.^20^^,^^21^^,^^23^^,^^27^^–^^29^^,^^31^^,^^33^^–^^36^^,^^38^^,^^39^ As the definitions of pregnancy loss outcomes can overlap, we aligned with the wording used within individual studies. The most commonly reported pregnancy loss was stillbirth (*n* = 7),^20^^,^^21^^,^^28^^,^^29^^,^^34^^,^^35^^,^^39^ with observed rates from 1.6%^34^ to 11.6%,^29^ whereas the least reported pregnancy loss was spontaneous abortion (*n* = 3).^31^^,^^34^^,^^38^ The rate of each pregnancy loss was fairly consistent (DIU [*n* = 6] ranged from 0.0%^35^ to 6.6%,^21^ miscarriage [*n* = 6] ranged from 1.0%^21^ to 8.0%,^28^ and spontaneous abortion [*n* = 2] ranged from 1.7%^38^ to 5.1%^31^). An increased rate of pregnancy loss among dengue-positive pregnancies was observed, but statistical testing provided mixed results. No studies explored the association between dengue severity and pregnancy loss.

Postpartum hemorrhage was reported in 10 studies.^20^^,^^21^^,^^23^^–^^25^^,^^33^^,^^34^^,^^36^^,^^38^^,^^39^ There was an unclear association between dengue-positive pregnancies and postpartum hemorrhage across all studies, ranging from 0.0%^24^^,^^38^ to 25.0%.^20^ Interpartum hemorrhage was not reported in any studies.

### Quality assessment.

Study quality was assessed using the AHFMR tool for quantitative studies (Supplemental Table 4). Only four studies were judged to be of the highest quality, sufficiently addressing all of the AHFMR tool questions (Table 4).^27^^,^^29^^,^^30^^,^^32^ Another four studies either partially addressed or did not address only one or two of the questions.^18^^,^^25^^,^^38^^,^^40^ Most of the studies were of lower quality and only partially addressed or did not address at least three of the questions of the AHFMR tool.^19^^–^^21^^,^^23^^,^^24^^,^^28^^,^^31^^,^^33^^–^^37^^,^^39^ The two studies that ranked as being the lowest quality partly addressed four questions and did not address three questions.^22^^,^^26^

## DISCUSSION

Among the 23 included studies, this review shows the increased burden that dengue-positive pregnant people face as compared with dengue-negative pregnant people. Of note, dengue-infected pregnant people were more often reported to present dengue infections during the third trimester of gestation and face an increased risk of maternal mortality, pregnancy loss, and preterm birth. However, data from the included studies presented heterogenous results, likely because of inconsistencies across methods of dengue detection and classification of outcomes.

Many pregnancy outcomes were well captured in this review. A notable trend was the higher rate of maternal mortality, which ranged from 0% to 21.7%.^18^^,^^20^^,^^24^^,^^25^^,^^28^^,^^33^^–^^35^^,^^37^^,^^38^^,^^40^ In comparison, the global dengue case fatality rate (CFR) in 2024 was about 0.065%, the year with the greatest number of cases to date.^41^^–^^43^ Furthermore, the estimated global proportion of deaths among women of reproductive age because of maternal causes from 2000 to 2020 was estimated to be 9.8%.^44^ Although these rates fall within our observed range, four included studies reported a higher maternal mortality rate.^18^^,^^20^^,^^36^^,^^37^ The work of Adam et al.^18^ was a high-quality study conducted in Sudan between 2008 and 2009. This study reported a maternal mortality rate of 21.7% (17 deaths of 78 dengue-infected pregnant women).^18^ Over the same 2-year period in Sudan, WHO Eastern Mediterrean Region reported ∼1,000 suspected dengue cases.^45^ Therefore, approximately 7.8% (*n* = 78/1,000) of the suspected dengue cases between 2008 and 2009 in Sudan were represented by the pregnant people in the study of Adam et al.,^18^ and 1.7% (*n* = 17/1,000) of the suspected cases were represented by mothers who died, which is higher than the global CFR. In addition, Tougma et al.^38^ found that mortality was significantly higher in the dengue-positive group than in the dengue-negative group (4.1% versus 0.3%, respectively). There are some physiological and immunological mechanisms that could explain the higher mortality rates among pregnant mothers infected with dengue. Immunologic fluctuations during pregnancy can compromise viral clearance as a means to protect the fetus, in turn causing increased severity of infection.^46^^–^^48^ Among dengue patients, those who experience severe dengue typically have higher viraemia, which should cause a stronger immunologic response and subsequently, lead to severe sequela and symptoms.^49^ But, as the mother’s immunological ability to clear viral loads is compromised and viremia increases, development of a severe dengue infection with consequential sequala or death outcomes would be expected. In our review, dengue severity was often reported within patient baseline characteristics, but mixed reported proportions of severe/nonsevere dengue across studies made it difficult to draw conclusions about the impact of dengue severity on outcomes. From the few studies that had reported ICU admission rates, observed mothers always presented with severe dengue.^24^^,^^35^^,^^36^^,^^40^ Evidence concerning other infectious diseases describes that pregnant people are at a greater risk of severe infection and death, including influenza, hepatitis E, herpes simplex virus, and malaria to name a few.^46^ Moreover, infection severity may be increased in pregnant people because of the many physiological changes experienced during pregnancy, including decreased lung capacity, urinary stasis, and changes in blood flow.^46^ In contrast, the maternal mortality rates reported in this review in addition to the other pregnancy outcomes may be confounded by the level of care and access to health care services available to patients as health systems in areas of low access may be unable to handle pregnancy complications. As the majority of studies were conducted in countries considered to be low-income and low-middle-income countries (*n* = 16), this makes it difficult to understand if pregnancy outcomes are because of access and quality of health services or because of dengue infection severity.^50^^,^^51^

Another evident pregnancy outcome was related to pregnancy loss. Thirteen studies reported at least one type of pregnancy loss (i.e., stillbirth, miscarriage, spontaneous abortion, or DIU). Notably, a numeric trend indicating an increase in pregnancy loss after dengue infection was observed but with mixed statistical results. For most types of pregnancy loss, dengue did not increase the risk of the event, except among stillbirths. Stillbirth was the most reported pregnancy loss type (*n* = 7),^20^^,^^21^^,^^28^^,^^29^^,^^34^^,^^35^^,^^39^ with a reported rate ranging from 1.6%^34^ to 11.6%^29^ among affected mothers. The United Nations Children’s Fund reported in 2021 that the rate of stillbirths was 1 in 72 newborns (1.4%),^52^^,^^53^ less than the range observed in this review. The existing literature describes that viral infections within the maternal–fetal interface can affect placental function, often resulting in a variety of complications, such as miscarriage, intrauterine growth restriction, and preterm birth. These complications are usually owing to the induced immunological response of viral recognition by the mother’s immune cells. Infections of the decidua, the amniotic fluid, or the placenta itself could elicit the production of inflammatory cytokines, which could lead to placental damage and miscarriage or preterm labor.^46^^,^^54^^–^^56^ In fact, preterm birth was another notable pregnancy outcome in this review. One major study reported that the global preterm birth rate between 2010 and 2020 was 9.8–9.9%.^57^ These rates fall within the preterm birth rates range captured by this review of 4.3–37.5%.^27^^,^^20^ However, 17 studies reported a rate of 10% or higher.^18^^–^^31^^,^^33^^,^^34^^,^^36^^,^^38^^–^^40^ Three^19^^,^^33^^,^^38^ of seven studies^19^^,^^22^^,^^26^^,^^27^^,^^33^^,^^38^^,^^39^ that assessed statistical differences found that the rate of preterm birth was significantly higher among dengue-positive pregnancies versus dengue-negative controls. One study investigated the placentas of dengue-infected pregnant women and reported deciduitis, coriodeciduitis, intervillositis, villositis, hypoxia, and erythrocyte sickling in the placental intervillous space among infected mothers. This describes how DENV can disrupt the maternal–fetal interface and placenta. Fetal damage and developmental complications can also occur even if the virus does not reach the fetus.^56^ Other pregnancy outcomes that were explored in this review (delivery type and postpartum hemorrhage) did not show any clear associations or trends with dengue infections.

Weeks 20–34 of gestation were most frequently reported as the time point of infection, with the proportion of patients ranging from 33%^38^ to 84%.^20^ Sinha and Datta^37^ found that 10.2% of patients infected in late pregnancy died, whereas no deaths were reported among those infected earlier in gestation. Nascimento et al.^30^ described that dengue infection in the third trimester increased the likelihood of women experiencing preterm births (adjusted OR: 1.24, 95% CI: 0.97–1.6, *P* = 0.08) as compared with noninfected pregnant women. Similarly, Agarwal et al.^34^ reported a high rate of preterm birth among mothers infected within the third trimester, and 41% of dengue-positive pregnant people diagnosed at 26–36 weeks of gestation experienced preterm birth (*n* = 9/22). Contraction of DENV infection and increased severity in the later stages of pregnancy may be because of the reductions of important immune cells during the late second and third trimesters, including B cells, natural killer cells, and complementary determining region T cells, which in turn, are hypothesized to reduce the production of interferon-y, tumor necrosis factor, and interleukin-6.^47^^,^^54^^,^^58^^–^^60^ Therefore, once the mother is infected during the later stages of pregnancy, viral clearance can become compromised because of this anti-inflammatory phase of gestation.^47^^,^^59^

Generally, epidemiology data were not well reported. Incidence and general hospitalization rates were not reported in any studies, and minimal data regarding dengue prevalence were found. Two studies did report period prevalences of dengue among pregnant people. Adam et al.^18^ reported that 0.7% of pregnant women (*n* = 78/10,820) had confirmed dengue in Sudan between 2008 and 2009, whereas Annan et al.^32^ reported that 5.2% of dengue-infected women (*n* = 4,943/94,832) in Mexico between 2012 and 2020 were pregnant; of those, 34.6% (*n* = 1,707) of cases were severe dengue.^32^ Overall, because of the lack of epidemiological data, the ability to draw meaningful conclusions was compromised. Given the differences in dengue patterns across countries, regions, and time frames, these findings are unlikely to accurately reflect the burden of dengue among pregnant individuals in other geographies. The lack of epidemiological data for dengue in pregnancy constitutes a key evidence gap and highlights a need for further research.

This SLR was performed with high technical standards in line with the best-practice systematic methods recommended by the *Cochrane Handbook for Systematic Reviews of Interventions*.^61^ In addition, this review is one of the few systematic reviews that holistically describes the burden of dengue infection in pregnant individuals. Evidence was identified for a variety of pregnancy outcomes; however, results generally ranged widely, and studies were highly heterogenous, of mixed quality, and associated with limitations. Studies were conducted in a range of different countries, used varied (and often poorly reported) methods to diagnose dengue, lacked consistent definitions for outcomes, and recruited widely different numbers of patients. Only four studies performed well across all domains of the quality assessment, with all others having limitations in at least one area. Many studies did not account for or adequately report confounding factors, such as access to obstetric and gynecologic health services, and reporting of variance measures was limited. As a result, the findings from this SLR may not be generalizable to all pregnant populations and should be interpreted with caution. Furthermore, the burden of dengue within specific regions is known to fluctuate over time. The studies identified in this SLR had different periods of data collection, many of which could now be considered outdated.

## CONCLUSION

The findings of this SLR lend weight to the increased risk of dengue during pregnancy, particularly regarding maternal mortality and pregnancy loss. Of note, the third trimester may be a more vulnerable time period not only for mothers to contract DENV but also, for increased severity because of infection. Lastly, more conclusive research efforts are needed to evaluate the risk of preterm birth among dengue-infected mothers as there may be a greater risk. The lack of epidemiological evidence concerning dengue-positive mothers compared with a moderate volume of data regarding pregnancy highlights a key evidence gap in the literature. The largely heterogeneous data captured by this review emphasizes the need for standardization of methods and disease classification regarding dengue infection. Further standardized quantitative comparisons of outcomes in dengue-positive and dengue-negative pregnant people will be particularly valuable in determining clear trends in the risk and burden of dengue during pregnancy.

Continued efforts to include pregnant people in dengue studies must persist. Vertical transmission of DENV is known to occur and can place the mother and fetus at risk.^62^ Vaccines to prevent DENV infection are currently available; however, they are contraindicated for pregnant women, leaving them vulnerable to infection and the potential for serious consequences for both themselves and their fetuses. As innovations in treatment and prevention progress, it is crucial to consider the needs of this population. With the expansion of transmission boundaries and the increased intensity of dengue epidemics because of climate change, it is anticipated that the incidence of DENV infection among pregnant women will rise.

## Supplemental Materials

10.4269/ajtmh.25-0311 Supplemental Materials
