# Supplementary material for: Burden of Dengue in Pregnant Individuals: A Systematic Literature Review
Source: Am J Trop Med Hyg. 2026 Apr 21;114(6):1032–43. doi: 10.4269/ajtmh.25-0311 (PMC13235611; doi:10.4269/ajtmh.25-0311)
Supplement: Supplemental Materials [file tpmd250311.SD1.pdf]

Supplementary Information

Supplementary methods

Search strategy

Search terms for the electronic databases included combinations of free-text and Medical Subject Heading (MeSH) or Emtree terms, grouped into the following categories:

- Disease area: Dengue + Pregnancy
- Study design terms: Epidemiologic studies, Observational study, Cohort Studies, Case-control Studies, Cross-Sectional Studies, Cross-sectional Studies, Clinical Studies, Follow-up Studies, Longitudinal Studies, Retrospective Studies and Prospective Studies
- Exclusion terms: studies indexed as including animals only, case reports, case studies, and editorials (MEDLINE/Embase only)
- Limits: 2010 onwards

Through the electronic database searches, a total of 625 publications were identified. After the removal of duplicates, 406 title/abstracts were reviewed against the pre-specified eligibility criteria (**Table 1**). Subsequently, 107 publications were considered potentially relevant for inclusion and underwent full-text review. A total of 42 publications were ultimately included from the electronic database searches. Through the supplementary hand searches, total of 1,456 publications were identified. Of these, six records were ultimately included in the SLR.

Full details of the search strategy for the electronic database searches, including all elements from the PRISMA reporting checklist,[1] are presented in **Supplementary Table 1**, **Supplementary Table 2** and **Supplementary Table 3**.

Supplementary results

Demographic characteristics

Age

Among the 16 studies that reported age for dengue-positive pregnant people (**Supplementary Figure 4**), mean or median age ranged from 23[2] to 32.65 years.[3] Two studies reported age for different subgroups

(Machain-Williams 2018 and Sinha 2023). Machain-Williams reported median age for three subgroups of dengue severity (dengue with without warning signs (n=54), dengue with warning signs (n=15), severe dengue (n=13); age ranges were similar across subgroups with no statistically significant difference reported ($p \geq 0.05$).[4] Sinha 2023 reported mean age for two subgroups of timepoint of infection (infection during early pregnancy [≤24 weeks] and infection during late pregnancy [>24 weeks]). The statistical difference between subgroups was not reported, however the 95% confidence interval (CI) of the two subgroups did overlap (infection during early pregnancy [≤24 weeks]: 95% CI [24.83, 30.77]; infection during late pregnancy [>24 weeks]: 95% CI [29.82, 34.53]).[3]

Nguyen 2021 reported data for laboratory-confirmed dengue in 32 neonates born of dengue-positive mothers. The median age at diagnosis was seven days (IQR: 6, 10); the median day of illness at diagnosis of dengue was two days (IQR: 1,4).[5]

Ethnicity

Three studies reported the ethnicity of included patients.[6, 7, 8]. In the Laos-based study, the majority of participants were Lao loum (97%),[6] in the French Guiana-based study, the majority were Maroon (56%)[7] and in the Brazil-based study, the majority were Pardo in all three subgroups (47–49%), followed by White (41–43%).[8] In this study, the proportions of different ethnicities were numerically well-balanced across the three subgroups, which were based on the trimester of infection.

Neonatal outcomes

Pregnancy complications

Intrauterine growth restriction (IUGR) was reported in five studies.[2, 9, 10, 11] Two were conducted in India,[9, 10] one in Cuba,[11] one in Thailand,[2] and one study did not report geography.[12] Pre-eclampsia was reported in seven studies; two from India,[10, 13] one each from Colombia,[14] Cuba,[11] French Guiana,[15] Mexico,[4] and Thailand.[2] There was an unclear association between dengue-positive pregnancies IUGR (n=5) and pre-eclampsia (n=7 studies), ranging from 2.1%[3]–10%[2, 10, 11] and 0%[11, 15]–7.7%,[14] respectively. Although, one study did investigate the severity of pre-eclampsia and dengue infection.[2] Severe pre-eclampsia occurred more often than mild pre-eclampsia (3.4% vs 0.8%), and was significantly higher in dengue-positive than dengue-negative pregnant people (3.4% vs 1%; $p=0.035$).

Neonatal mortality

Twenty-eight-day neonatal mortality

Six studies conducted in Asia,[2, 5, 9, 10, 16, 17] reported 28-day neonatal mortality, which ranged from 0%[5, 16, 17] to 5%.[10] Overall, three studies reported that no neonatal deaths occurred within 28 days of birth,[5, 16, 17] one Thai study reported one neonatal death within 28 days of birth,[2] and two studies (both conducted in India) reported two neonatal deaths within 28 days of birth.[9, 10] Two studies conducted in India (Sagili 2022)[9] and Thailand (Singkibutr 2022)[2] presented statistical analyses comparing the rate of 28-day neonatal mortality between newborns born of dengue-positive versus newborns born on dengue-negative mothers; no statistical difference was found in either study. Notably the dengue-negative comparator populations differed slightly; Sagili 2022 used a population of patients who were admitted to hospital with fever while Singkibutr 2020 used a more general population of dengue-negative hospitalized pregnant people.[2, 9]

Perinatal mortality

One study, Adam 2010, a retrospective cohort study that reviewed all deliveries at two hospitals in eastern Sudan between 2008–2009, reported the rate of perinatal mortality, defined as the number of deaths of newborns born at ≥28 weeks of gestation until the end of day seven. Seven (8.9%) perinatal deaths occurred.[18]

Birthweight and size

Fourteen studies reported birthweight.[2, 4, 5, 6, 7, 8, 9, 10, 12, 14, 15, 16, 18, 19] Birthweight was reported inconsistently across studies, with studies reporting mean birthweight, categorical ranges of birthweight or ‘low birthweight’ which was not defined further.

Five studies reported mean birthweight,[5, 9, 15, 16, 19] which ranged from 2,442.8 g (standard deviation [SD] 720.0 g)[9] to 3,280 g (490.0 g);[5] SDs were generally high across all estimates. Three studies reported median birthweight, which ranged from 2,865 g[2] to 3,200 g.[15] However, one of these studies[6] reported a median birthweight of 3,000 g and a range of 3,320–3,900 g for newborns born to mothers with dengue; these data should be treated with caution. Of note, the range of median birthweights reported across studies (335 g) was smaller than the range of mean birthweights (838 g), as calculated from the median and mean birthweights reported in the extracted studies.

Six studies reported on newborns with birthweight <2,500 g;[2, 6, 7, 8, 9, 18] the proportion of newborns reported for this weight category ranged from 0%[6] to 46.5%.[9] Both Singkibutr 2020 and Sagili 2022 investigated statistical significance between newborns born of dengue-positive and dengue-negative mothers; no statistically significant difference was observed in either study for the proportion of newborns with birthweight <2,500 g (p=0.454 and p=0.166, respectively).[2, 9] Nascimento 2017 reported birthweight across varying timepoints of infection during pregnancy. The proportion of newborns with birthweight <2,500 g was similar between those born to mothers infected during either the first (8.9%) or third trimester (7.8%).[8] This study was judged to be of the highest quality (**Table 4**).

Machain-Williams 2018, a retrospective cohort of 82 patients in Mexico, explored the relationship between dengue severity and birthweight. In all subgroups (newborns of mothers infected with dengue without warning signs, newborns of mothers infected with dengue with warning signs and newborns of mothers infected with severe dengue who died), the birthweight of all newborns was 2,500–4,000 g.[4]

Four studies reported low birthweight, which was not defined further.[4, 10, 12, 14] The proportion of newborns with low birthweight ranged between 20%[4] and 100%.[12] The study that reported the lowest rate of low birthweight (Machain-Williams 2018) was a retrospective cohort analysis drawing from a regional Mexican sample. This finding may not be broadly generalizable as it was measured specifically in a subgroup of five patients diagnosed with severe dengue who died between the 1st and 10th day post-partum.[4]

Small for gestational age (SGA), defined as birthweight <10th percentile for gestational age, was reported in two studies; 7/72 (9.7%; Basurko 2018)[15] and 29/88 (33.0%, Sagili 2022)[9] newborns born of dengue-positive mothers were reported as being SGA. Please note that percentages were manually calculated from data available within the study. These studies were judged to be of the highest and moderate quality, respectively (**Table 4**). Neither study observed a statistically significant difference in SGA between newborns of mothers admitted to hospital with fever who tested positive for dengue or who tested negative for dengue (Basruko 2018: p=0.729; Sigali 2022: p=0.055).[9, 15]

Major congenital abnormalities

Two studies conducted in Asia, Mulyana 2020 and Nguyen 2021, reported that no major congenital abnormalities occurred in newborns of dengue-positive mothers among 41 and 32 births respectively. These

studies were judged to be of low and moderate quality, respectively (**Table 4**). One study, Nascimento 2017, reported a total of 27 congenital abnormalities among 3,789 births (0.7%); abnormalities were defined as malformation according to the International Classification of Disease, 10th revision (ICD-10). This study drew the study population from multiple centers within Brazil via the Brazilian national reportable disease information system database and was judged to be of the highest quality (**Table 4**). It was the only study to investigate the statistical difference in major congenital abnormalities between newborns born of dengue-positive mother and dengue-negative mothers. No statistically significant difference in major congenital abnormalities was found between dengue-positive mothers compared against dengue-negative mothers (p=0.51).[8]

Vertical transmission of dengue infection

Vertical transmission of dengue from pregnant people to their newborns was reported in seven studies. Of the five studies that defined vertical transmission, the majority (n=4) determined vertical transmission based on laboratory testing; one study (Mulyana 2020) used both clinical and laboratory evaluation of dengue. This study was judged to be of the lowest quality (**Table 4**).

The rate of vertical transmission ranged from 0%[20] to 76.5%.[14] One study, Muralidhar 2021 a retrospective cohort study of 35 dengue-positive pregnant people and their newborns, reported no cases of vertical transmission, however, this study was judged to be of the lowest quality (**Table 4**). The authors also did not report how vertical transmission was defined.[20] The highest rate of vertical transmission was reported by Restrepo Jaramillo 2013,[14] with IgG antibodies being identified in 26 newborns of dengue-positive pregnant people vertical. Transmission of dengue was identified using ELISA.[15] This study was judged to be of moderate to low quality (**Table 4**).

Supplementary Tables

Supplementary Table 1. Search terms for use in MEDLINE (to be searched via the OvidSP platform)

Term Group	#	Searches	Hits (22nd November 2023)
	1	exp Dengue/ or *Mosquito-Borne Diseases/ or *Arbovirus Infections/ or *Flavivirus Infections/	19183
	2	(dengue$ or DENV or breakbone$ or break-bone$ or dandy$ or bouquet$ or solar fever$ or sun fever$).ti,ab,kf.	31550
**Population**	3	1 or 2	34781
	4	exp Pregnancy/ or (pregnan$ or gestation$ or prenatal$ or pre-natal$ or antenatal$ or ante-natal$ or maternal$ or neonat$ or fetal or fetal or miscarriage).ti,ab,kf.	1627284
	5	3 and 4	1566
	6	Epidemiologic Studies/	9436
	7	Observational Study/	149157
	8	Cohort Studies/	334920
	9	exp Case-Control Studies/	1461115
	10	Cross-Sectional Studies/	484864
	11	Clinical Study/	5720
	12	Follow-Up Studies/	694310
	13	Longitudinal Studies/	168134
**Observational and real-world studies**	14	Retrospective Studies/	1160553
	15	Prospective Studies/ not Randomized Controlled Trials as Topic/	665661
	16	(observational adj (study or studies)).ti,ab,kf.	170824
	17	(cohort adj (study or studies)).ti,ab,kf.	337556
	18	cohort analy$.ti,ab,kf.	13269
	19	case control.ti,ab,kf.	159146
	20	cross sectional.ti,ab,kf.	537280
	21	(follow up adj (study or studies)).ti,ab,kf.	59209
	22	longitudinal.ti,ab,kf.	338565
	23	retrospective.ti,ab,kf.	776673

Term Group	#	Searches	Hits (22nd November 2023)
	24	(chart adj3 review$).ti,ab,kf.	55847
	25	exp Registries/	118652
	26	(registry or registries).ti,ab,kf.	184054
	27	(prospective adj (study or studies)).ti,ab,kf.	213875
	28	(epidemiologic$ adj (study or studies)).ti,ab,kf.	97510
	29	(evaluation adj (study or studies)).ti,ab,kf.	7707
	30	(medical record$ or real world or population based or survey$ or questionnaire$ or medicare or medicaid or marketscan).ti,ab,kf.	1791055
	31	or/6-30	5190172
**Exclusion**	32	exp Animals/ not exp Humans/	5173774
	33	(comment or editorial or case reports or historical article).pt.	4193740
	34	Editorial/	672702
	35	(case stud$ or case report$).ti.	394562
	36	or/32-35	9362992
	37	5 and 31	359
	38	37 not 36	334
**Combination**	39	limit 38 to yr="2010-current"	266
	40	remove duplicates from 39	**265**

Database(s): Ovid MEDLINE(R) and Epub Ahead of Print, In-Process, In-Data-Review & Other Non-Indexed Citations and Daily 1946 to November 21, 2023.

132 **Supplementary Table 2. Search terms for use in Embase (to be searched via the OvidSP platform)**

Term Group	#	Searches	Hits (22nd November 2023)
Population	1	exp dengue/ or *mosquito borne disease/ or *arbovirus infection/ or *flavivirus infection/	29477
	2	(dengue$ or DENV or breakbone$ or break-bone$ or dandy$ or bouquet$ or solar fever$ or sun fever$).ti,ab,kf.	39694
	3	1 or 2	44768
	4	exp pregnancy/ or (pregnan$ or gestation$ or prenatal$ or pre-natal$ or antenatal$ or ante-natal$ or maternal$ or neonat$ or fetal or fetal or miscarriage).ti,ab,kf.	1775719
	5	3 and 4	2268
Observational and real-world studies	6	epidemiology/	246919
	7	observational study/	344904
	8	cohort analysis/	1074052
	9	exp case control study/	227879
	10	cross-sectional study/	593721
	11	clinical study/	164660
	12	follow up/	2100643
	13	longitudinal study/	200299
	14	retrospective study/	1519303
	15	prospective study/ not "randomized controlled trial (topic)"/	883171
	16	(observational adj (study or studies)).ti,ab,kf.	263791
	17	(cohort adj (study or studies)).ti,ab,kf.	486577
	18	cohort analy$.ti,ab,kf.	20166
	19	case control.ti,ab,kf.	210564
	20	cross sectional.ti,ab,kf.	699660
	21	(follow up adj (study or studies)).ti,ab,kf.	78507
	22	longitudinal.ti,ab,kf.	457816
	23	retrospective.ti,ab,kf.	1285073
	24	(chart adj3 review$).ti,ab,kf.	118593
	25	exp disease registry/	68763

Term Group	#	Searches	Hits (22nd November 2023)
	26	(registry or registries).ti,ab,kf.	304292
	27	(prospective adj (study or studies)).ti,ab,kf.	327916
	28	(epidemiologic$ adj (study or studies)).ti,ab,kf.	126632
	29	(evaluation adj (study or studies)).ti,ab,kf.	10537
	30	(medical record$ or real world or population based or survey$ or questionnaire$ or medicare or medicaid or marketscan).ti,ab,kf.	2473739
	31	or/6-30	7843389
	32	("conference abstract" or "conference review").pt.	4971329
	33	limit 32 to yr="1974-2020"	4165402
	34	exp animal/ not exp human/	5170086
**Exclusion**	35	(comment or editorial or case reports or historical article).pt.	786350
	36	editorial/ or case report/	3689313
	37	(case stud$ or case report$).ti.	477442
	38	or/33-37	12500547
	39	5 and 31	645
**Combination**	40	39 not 38	419
	41	limit 40 to yr="2010-current"	361
	42	remove duplicates from 41	**357**

Database(s): Embase 1974 to November 6, 2023.

Supplementary Table 3. Search terms for use in the CDSR (to be searched via the Wiley platform)

Term Group	#	Searches	Hits (22nd November 2023)
**Population**	1	[mh Dengue] or [mh ^"Mosquito-Borne Diseases"] or [mh ^"Arbovirus Infections"] or [mh ^”Flavivirus Infections”]	450
	2	(dengue* or DENV or breakbone* or (break NEXT bone*) or dandy* or bouquet* or (solar NEXT fever*) or (sun NEXT fever*)):ab,ti,kw	920
	3	#1 OR #2 with Cochrane Library publication date Between Jan 2010 and Dec 2023, in Cochrane Reviews	**3**

Database(s): CDSR, Issue 11 of 12, November 2023.

Supplementary Table 4. AHFMR questions

Question number	Question
1	Question/objective sufficiently described
2	Study design evident and appropriate
3	Method of subject/comparison group selection or source of information/input variables described and appropriate?
4	Subject (and comparison group, if applicable) characteristics sufficiently described?
5	If interventional and random allocation was possible, was it described?
6	If interventional and blinding of investigators was possible, was it reported?
7	If interventional and blinding of subjects was possible, was it reported?
8	Outcome and (if applicable) exposure measure(s) well defined and robust to measurement/ misclassification bias? Means of assessment reported?
9	Sample size appropriate?[a]
10	Analytic methods described/justified and appropriate?
11	Some estimate of variance is reported for the main results?
12	Controlled for confounding?
13	Results reported in sufficient detail?
14	Conclusions supported by the results?

[a]Sample size was considered appropriate (“Y”) if the study sample was drawn from a regional or national population. Sample size was considered partially appropriate (“P”) if the study sample was drawn from a less-than-regional scope (e.g. from an individual hospital or city) and ≥30 participants were recruited. AHFMR: Alberta Heritage Foundation for Medical Research. Source: Kmet et al. 2004.[21]

Supplementary Figures

Eligibility flowcharts used to aid review of title and abstract and full-text articles are presented in **Supplementary Figure 1** and **Supplementary Figure 2** respectively.

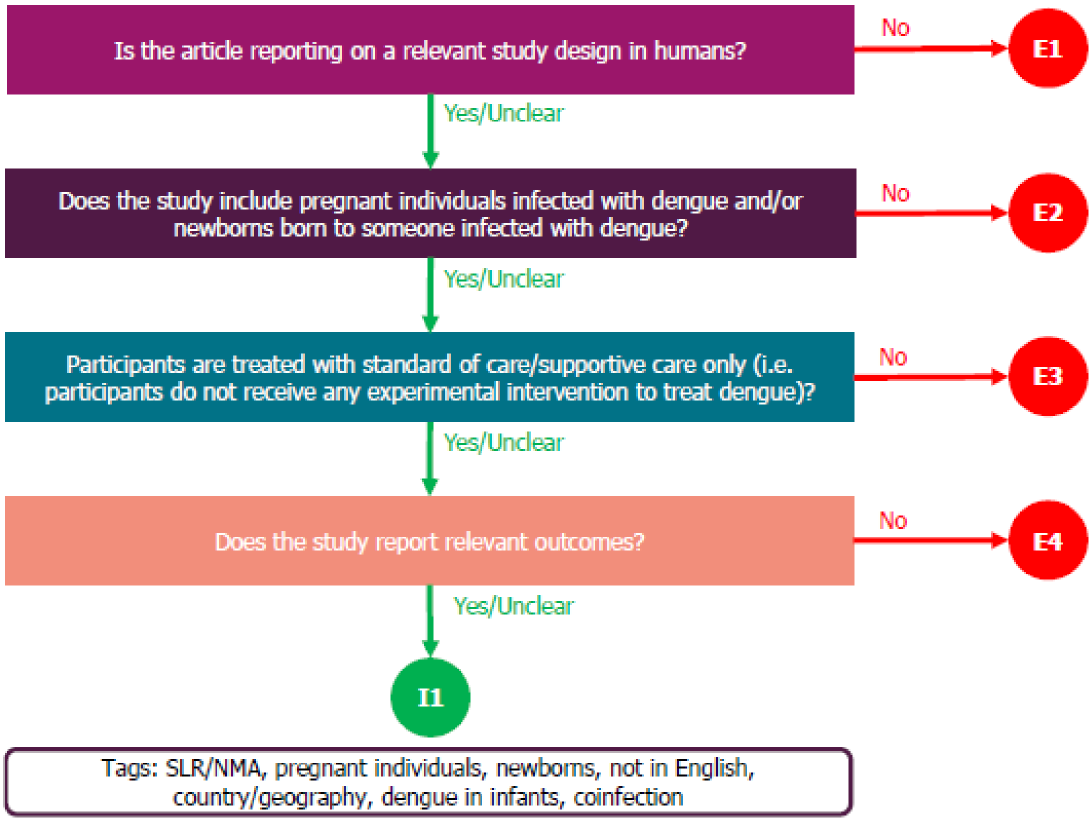

Supplementary Figure 1. Eligibility flowchart for the title/abstract review stage

I# (where '#' denotes a number) indicates an inclusion category, while E# (where '#' denotes a number) indicates an exclusion category.

SLR: systematic literature review; NMA: network meta-analysis.

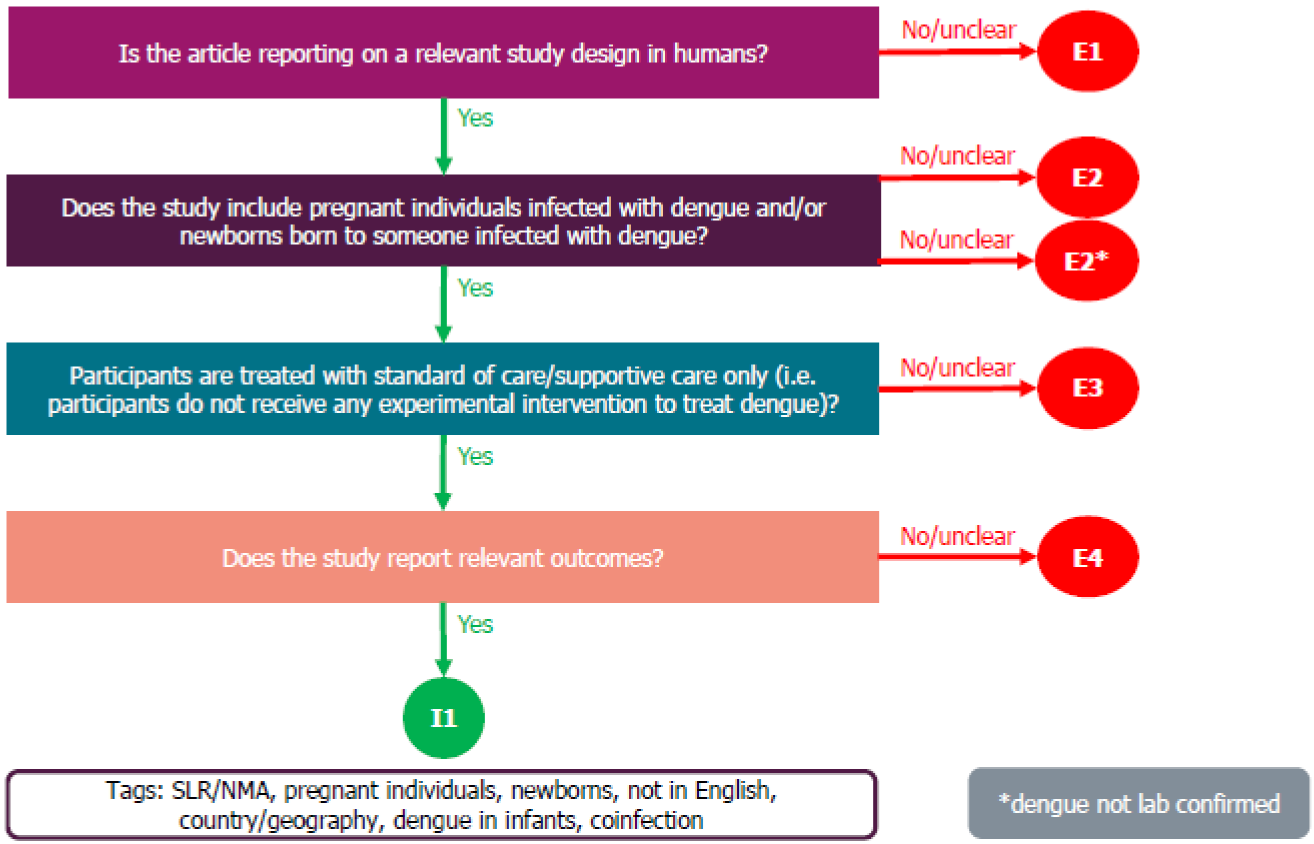

Supplementary Figure 2. Eligibility flowchart for the full-text review stage

I# (where '#' denotes a number) indicates an inclusion category, while E# (where '#' denotes a number) indicates an exclusion category.

SLR: systematic literature review; NMA: network meta-analysis

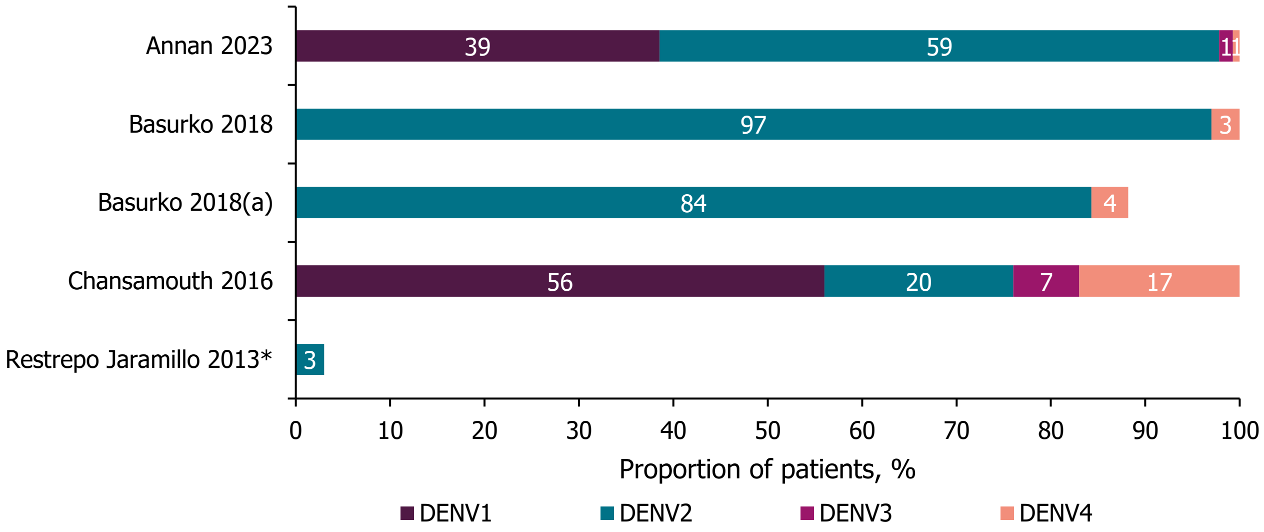

Supplementary Figure 3. Dengue serotype across extracted studies

Data were rounded to 0 decimal places, consistent with the highest level of granularity consistent across all included studies. Studies report dengue serotype for all dengue-positive pregnant people, unless otherwise indicated. Restrepo Jaramillo 2013 primarily use ELISA testing to determine dengue infection, however a single patient was tested using PCR allowing for the reporting of dengue serotype within this single patient.[14] *Percentages were manually calculated from data available within the study.

DENV1: dengue virus one; DENV2: dengue virus two; DENV3: dengue virus three; DENV4: dengue virus four.

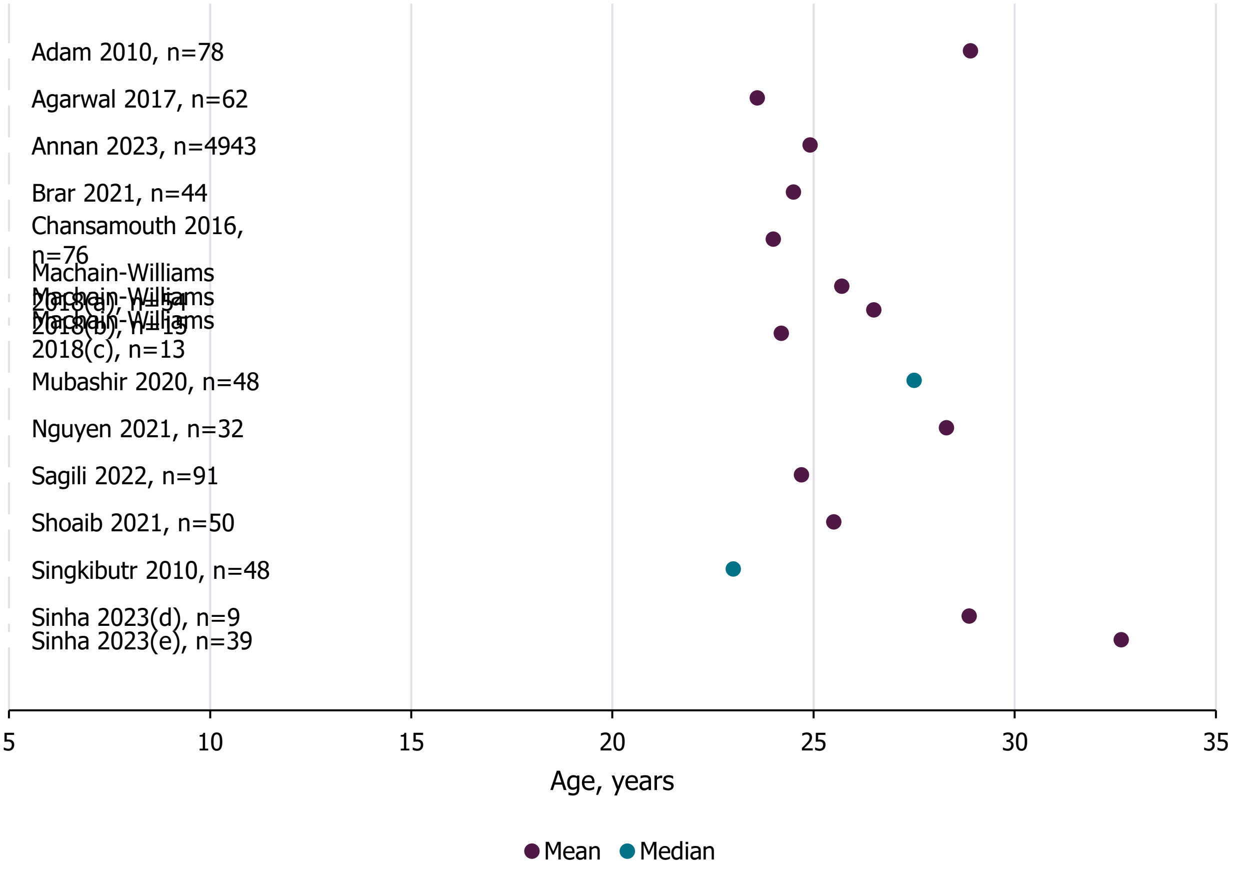

Supplementary Figure 4. Age across extracted studies

Seven of the included studies reported age categorically. These datapoints have been omitted from the figure due to the inability to determine an accurate mean or median from the reported data.[3, 5, 7, 8, 15, 16, 22] (a)Dengue without warning signs; (b)dengue with warning signs; (c)severe dengue; (d)dengue infection during early pregnancy (≤24 weeks); (e)dengue infection during late pregnancy (>24 weeks).

Supplementary References

1. Rethlefsen ML, Kirtley S, Waffenschmidt S, Ayala AP, Moher D, Page MJ, Koffel JB, 2021. PRISMA-S: an extension to the PRISMA statement for reporting literature searches in systematic reviews. Systematic reviews 10: 1-19.
2. Singkibutr T, Wuttikonsammakit P, Chamnan P, 2020. Effects of dengue infection on maternal and neonatal outcomes in Thai pregnant women: A retrospective cohort study. Journal of the Medical Association of Thailand 103(2): 155-162.
3. Sinha R, Datta MR, 2023. Dengue in Early Pregnancy: A Neglected Problem? Cureus 15: e38740.
4. Machain-Williams C, Raga E, Baak-Baak CM, Kiem S, Blitvich BJ, Ramos C, 2018. Maternal, Fetal, and Neonatal Outcomes in Pregnant Dengue Patients in Mexico. Biomed Res Int 2018: 9643083.
5. Nguyen TM, Huan VT, Reda A, Morsy S, Nam Giang HT, Tri VD, Mau NK, Elfaituri MK, Hieu TH, Hung NT, Hirayama K, Huy NT, 2021. Clinical features and outcomes of neonatal dengue at the Children's Hospital 1, Ho Chi Minh, Vietnam. 1: 104758.
6. Chansamouth V, Thammasack S, Phetsouvanh R, Keoluangkot V, Moore CE, Blacksell SD, Castonguay-Vanier J, Dubot-Peres A, Tangkhabuanbutra J, Tongyoo N, Souphaphonh P, Sengvilaipaseuth O, Vongsouvath M, Phommasone K, Sengdethka D, Seurbsanith A, Craig SB, Hermann L, Strobel M, Newton PN, 2016. The Aetiologies and Impact of Fever in Pregnant Inpatients in Vientiane, Laos. PLoS Negl Trop Dis 10: e0004577.
7. Friedman EE, Dallah F, Harville EW, Myers L, Buekens P, Breart G, Carles G, 2014. Symptomatic Dengue infection during pregnancy and infant outcomes: a retrospective cohort study. PLoS Negl Trop Dis 8: e3226.
8. Nascimento LB, Siqueira CM, Coelho GE, Siqueira JB, Jr., 2017. Symptomatic dengue infection during pregnancy and livebirth outcomes in Brazil, 2007-13: a retrospective observational cohort study. Lancet Infect Dis 17: 949-956.
9. Sagili H, Krishna RS, Dhodapkar R, Keepanasseri A, 2022. Maternal & perinatal outcome of fever in pregnancy in the context of dengue - A retrospective observational study. Indian J Med Res 156: 619-623.
10. Brar R, Sikka P, Suri V, Singh MP, Suri V, Mohindra R, Biswal M, 2021. Maternal and fetal outcomes of dengue fever in pregnancy: a large prospective and descriptive observational study. Arch Gynecol Obstet 304: 91-100.
11. López Barroso R, Deulofeu Betancourt I, Fayad Saeta Y, Macías Navarro MM, Delgado Guerra G, 2010. Repercusión del dengue serotipo 3 sobre el embarazo y producto de la concepción. Revista Cubana de Obstetricia y Ginecología 36: 42-50.
12. Kaur K, Singh J, 2023. To investigate the maternal and foetal consequences of dengue fever in pregnant women. Journal of Cardiovascular Disease Research 14(3): 967-970.
13. Garg R, Malhotra N, Pathak A, Singh D, Agrawal P, Gautam A, Gupta P, 2021. Maternal and Perinatal Outcome in Dengue Fever in Pregnancy in North India. Journal of South Asian Federation of Obstetrics and Gynaecology 13: 412-414.
14. Restrepo Jaramillo BN, Isaza DM, Salazar CL, Ramirez JL, Ramirez RE, Alvarez G, 2013. Vertical transmission and transplacental antibody transfer in dengue infection. [Spanish]. Salud(i)Ciencia 20(1): 17-22.
15. Basurko C, Everhard S, Matheus S, Restrepo M, Hilderal H, Lambert V, Boukhari R, Duvernois JP, Favre A, Valmy L, Nacher M, Carles G, 2018. A prospective matched study on symptomatic dengue in pregnancy. PLoS One 13: e0202005.
16. Agarwal K, Malik S, Mittal P, 2017. A retrospective analysis of the symptoms and course of dengue infection during pregnancy. Int J Gynaecol Obstet 139: 4-8.
17. Mulyana RS, Pangkahila ES, Pemayun TGA, 2020. Maternal and Neonatal Outcomes during Dengue Infection Outbreak at a Tertiary National Hospital in Endemic Area of Indonesia. 1: 161-166.
18. Adam I, Jumaa AM, Elbashir HM, Karsany MS, 2010. Maternal and perinatal outcomes of dengue in PortSudan, Eastern Sudan. Virol J 7: 153.
19. Mubashir M, Ahmed KS, Mubashir H, Quddusi A, Farooq A, Ahmed SI, Jamil B, Qureshi R, 2020. Dengue and malaria infections in pregnancy : Maternal, fetal and neonatal outcomes at a tertiary care hospital. Wien Klin Wochenschr 132: 188-196.

20. Muralidhar L, Poojari AC, Bilal F, 2021. Clinical Study on Spectrum of Dengue Morbidity in Pregnancy and Its Impact. Journal of South Asian Federation of Obstetrics and Gynaecology 13: 251-253.
21. Kmet LM, Cook LS, Lee RC, 2004. Standard quality assessment criteria for evaluating primary research papers from a variety of fields.
22. Tougma SA, Zoungrana/Yameogo WN, Dahourou DL, Salou/Kagone IA, Compaore TR, Kabore A, Kagone T, Drabo MK, Meda N, 2020. Dengue virus infection and pregnancy outcomes during the 2017 outbreak in Ouagadougou, Burkina Faso: A retrospective cohort study. PLoS One 15: e0238431.
